# Research Advances in Mechanical Properties and Applications of Dual Network Hydrogels

**DOI:** 10.3390/ijms232415757

**Published:** 2022-12-12

**Authors:** Xuanjun Ning, Jiani Huang, Yimuhan A, Ningning Yuan, Cheng Chen, Donghai Lin

**Affiliations:** 1 School of Energy and Materials, Shanghai Polytechnic University, Shanghai 201209, China; 2 School of Materials and Metallurgy, University of Birmingham, Birmingham B15 2TT, UK; 3 Shanghai Engineering Research Center of Advanced Thermal Functional Materials, Shanghai Polytechnic University, Shanghai 201209, China

**Keywords:** double-network, hydrogel, mechanical properties

## Abstract

Hydrogels with a three-dimensional network structure are particularly outstanding in water absorption and water retention because water exists stably in the interior, making the gel appear elastic and solid. Although traditional hydrogels have good water absorption and high water content, they have poor mechanical properties and are not strong enough to be applied in some scenarios today. The proposal of double-network hydrogels has dramatically improved the toughness and mechanical strength of hydrogels that can adapt to different environments. Based on ensuring the properties of hydrogels, they themselves will not be damaged by excessive pressure and tension. This review introduces preparation methods for double-network hydrogels and ways to improve the mechanical properties of three typical gels. In addition to improving the mechanical properties, the biocompatibility and swelling properties of hydrogels enable them to be applied in the fields of biomedicine, intelligent sensors, and ion adsorption.

## 1. Introduction

Hydrogels have high hydrophilicity and high swelling, and the three-dimensional structure of their polymers, due to their porous structure, determines their excellent water absorption and swelling properties, their unique softness, ductility, and biocompatibility, and features the combination of biological tissue samples [1]. They are usually used in biomedical, intelligent sensors, ion adsorption, etc. However, the porous and heterogeneous structure, high swelling properties, and insufficient energy dissipation capacity of hydrogels also determine their poor mechanical properties. Hydrogels prepared from a single material are widely used in biological tissue engineering [2]. However, in other application fields, such as contact lenses [3], wound dressings [4], slow drug release [5], tissue scaffolds [6], and drug delivery carriers [7], low strength and toughness limit the development of hydrogels. Studies have shown that the common mechanical properties of hydrogels are as follows. First, the soft flexibility and muscular rigidity of the cross-linking points in the internal network of the hydrogel lead to the lack of a buffering effect in the hydrogel, and the hydrogel can be directly damaged by stress impact. Secondly, the flexible cross-linking force of hydrogels is poor, which makes it challenging to change the shape of hydrogels and difficult to recover after deformation. Thirdly, the lack of a dissipation mechanism in the hydrogel network makes the hydrogel unable to effectively disperse energy after being stressed, damaging the entire network. The high mechanical strength and toughness of hydrogel are keys to development; therefore, many scholars have put forward that a dynamic covalent bond and the stability of a non-covalent bond are needed to improve the toughness and mechanical strength of the hydrogel [5,6,7,8,9,10,11], for example, double network (DN) hydrogel, topology (TP) hydrogel and nanocomposite hydrogel (NC), hydrogen bond enhanced hydrogel, and macromolecular composite hydrogel microspheres.

The concept of DN hydrogel was first proposed by Gong [12] from Hokkaido University, Japan. Dual-network hydrogels have two different network structures, one is an electrolyte network structure with high cross-linking density but poor mechanical properties and the other is a neutral network structure with low or no cross-linking, which is used to fill the internal gap of the first structure [13]. Based on maintaining the original hydrogel properties, the dual-network system improved the strength and mechanical properties of hydrogels, which was also attributed to the internal fracture of covalent bonds in the first network, which dissipated energy and increased the resistance of cracks. This concept of a “sacrificial bond” was also proposed by the Gong team and verified with experiments.

**Figure 1:**
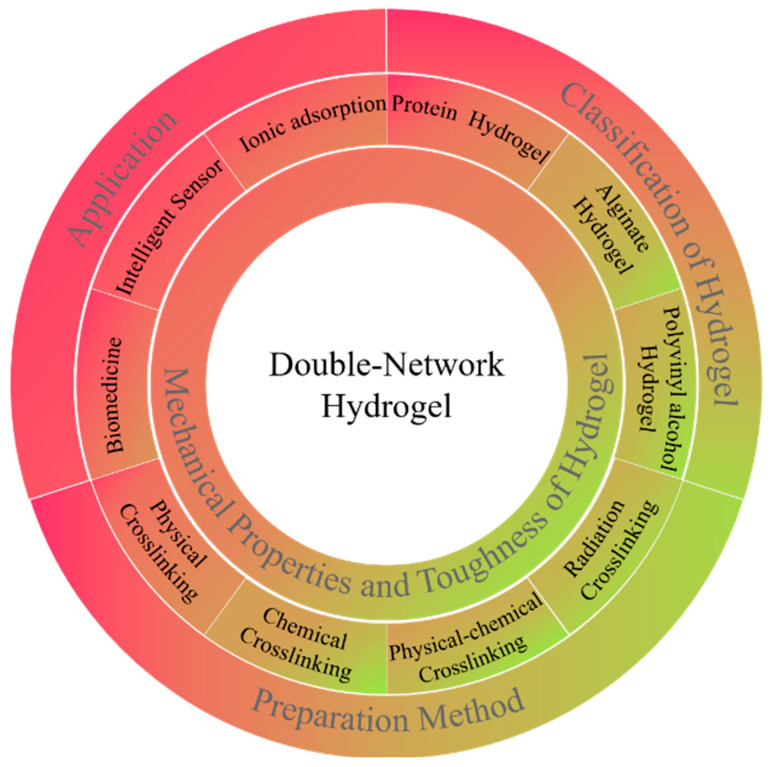
Research progress of double network hydrogel.

Up to now, there have been a variety of cross-linking methods (such as physical cross-linking, chemical cross-linking, physical–chemical cross-linking, radiation cross-linking, etc.) to prepare DN hydrogels. This review introduces the preparation methods of different hydrogels, the differences in mechanical properties, and the preparation of the mainstream hydrogel material (such as poly (vinyl alcohol) (PVA) and sodium alginate (SA) and proteins) in improving the mechanical properties, the progress in the research, and finally, lists the double network hydrogels in different fields of application, as shown in Figure 1.

## 2. Preparation Method of DN Hydrogel

In DN hydrogels, the first network provides high hardness and strength, which is also the key to determining the mechanical properties of the hydrogel. In contrast, the second network includes toughness [14] to ensure the suitable tensile property of the hydrogel. The two networks are cross-linking by physical non-covalent bonds or chemical covalent bonds. The entirely physical cross-linking hydrogel has excellent self-healing properties. Still, due to the formation of non-covalent bonds in the cross-linking process, its mechanical strength and mechanical stability are not as good as those of entirely chemical and physical–chemical cross-linking hydrogels. In the synthesis of entirely chemical and physical–chemical cross-linking hydrogels, the formation of internal solid covalent bonds improves the mechanical properties of hydrogels. However, the irreversible and permanent fracture of the network caused by chemical cross-linking makes it difficult for hydrogels to realize self-healing and self-repair. The following four cross-linking methods are compared, and each has advantages in specific applications. The differences in mechanical properties of the hydrogels prepared by different cross-linking methods are summarized in Table 1.

### 2.1. Physically Cross-Linking Hydrogels

**Figure 2:**
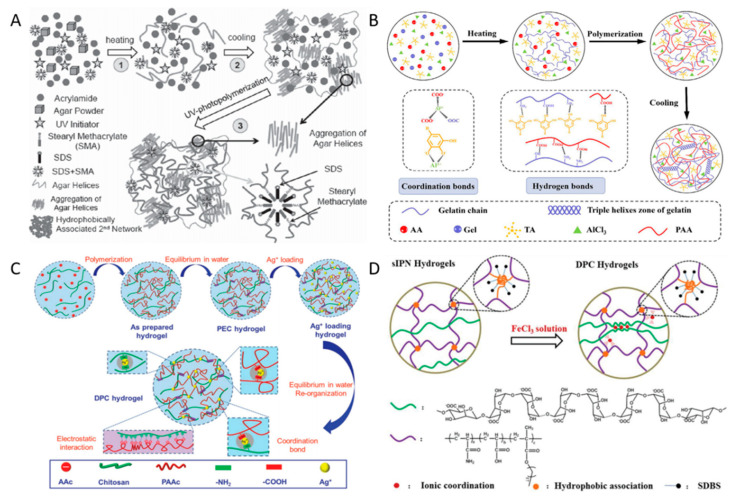
(**A**) Preparation mechanism of Agar/hydrophobic associated polyacrylamide (HPAAm) hydrogel. Reprinted with permission from [15]. 2015, Wiley; (**B**) Preparation mechanism of four-element DN physical cross-linking hydrogel. Reprinted with permission from [16]. 2022, Elsevier; (**C**) Preparation mechanism of PEC and DPC hydrogels. Reprinted with permission from [17]. 2018, Wiley; (**D**) Preparation mechanism of sodium alginate/polyacrylamide-acrylate-octadecyl methacrylate Fe^3+^ (SA/P(AAM-AAC-OMA)-Fe^3+^) hydrogel. Reprinted with permission from [22]. 2021, Wiley.

Physically cross-linking hydrogels are sensitive to external changes. In the process of cross-linking, the formation of reversible hydrogen bonds can cause the hydrogel to have good self-healing properties after damage. Chen [15] synthesized the fully physically cross-linking Agar/hydrophobic associated polyacrylamide (HPAAm) hydrogel using the one-pot method (Figure 2A). The experiment proved that introducing HPAAm in the second network improved the mechanical properties of the hydrogel by dissipating energy. The unique physical cross-linking reversible network structure in the hydrogel causes the hydrogel to have excellent self-healing properties. Bai [16] prepared a four-element DN physical cross-linking hydrogel using Gelatin (Gel), acrylic acid (AA), tannic acid (TA), and aluminum chloride (AlCl_3_), and the preparation mechanism is shown in Figure 2B. The first network was prepared with free radical polymerization of acrylic acid. The second network was formed using triple helical bundles stabilized by hydrogen bonds in the state of hydrogel freezing and permeated with the first network to finally form GATA (Gel, AA, TA, AlCl_3_) hydrogel. Due to the formation of reversible hydrogen bonds and coordination bonds, the maximum tensile strength of the hydrogel can reach 216 KPa, and the 3 h self-healing efficiency can be 88.4% at 60 °C.

Dual-physical cross-linking (DPC) DN hydrogel is composed of a network formed by two kinds of physical cross-linking. Cao [17] added acrylic acid to concentrated chitosan (CS) solution to polymerize to prepare polyelectrolyte complex (PEC) hydrogel (Figure 2C) and then introduced silver ion (Ag^+^) into it to obtain DPC hydrogel. High-density electrostatic interaction and the formation of coordination bonds between Ag^+^ and amino and carboxyl groups in PEC give the hydrogel strength up to 24 MPa and toughness up to 84.7 MJm^−3^. Yang [22], by combining reversible hydrophobic binding (HA) with low binding energy and ion coordination (IC) with high binding energy, prepared sodium alginate/polyacrylamide acrylate-octadecyl methacrylate Fe^3+^ (SA/P(AAM-AAC-OMA)-Fe^3+^) hydrogel (Figure 2D). The former provides high tensile properties, while the latter offers high tensile modulus and strength. The synergistic effect of the two offers good mechanical properties for DPC hydrogel. The tensile strength reaches 3.31 MPa, the tensile modulus is 0.65 MPa, and the toughness is 27.8 MJm^−3^.

### 2.2. Chemical Cross-Linking Hydrogels

**Figure 3:**
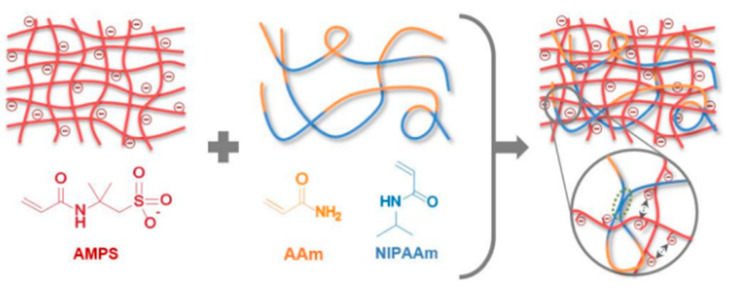
PAMPS/P(NiPAAm-Co-AAM) DN hydrogel cross-linking process. Reprinted with permission from [18]. 2019, American Chemical Society.

The network of covalent bonds in chemically cross-linking hydrogels provides mechanically solid strength. Still, when hydrogels break, the rupture of covalent bonds leads to permanent and irreversible damage to hydrogels. The hydrogel prepared by Means [18] uses poly (2-acrylamide-2-methylpropane sulfonic acid) (PAMPS) and poly (*N*-isopropyl acrylamide-co-polyacrylamide) [P (NiPAAm-co-AAM))] as the first and second networks of the hydrogel, as shown in Figure 3. Compared with the single-network hydrogel, when applied to cartilage, the compressive strength of the hydrogel can reach 25 MPa, 50 times that of the single-network hydrogel, which is formed entirely by chemical cross-linking. However, during stretching, the fracture of the covalent bond made the hydrogel unable to recover and appeared to soften, and the mechanical properties also decreased significantly. In addition, the toxic solvents such as glutaraldehyde [23], dye glycosides, and boric acid [24] used in the chemical cross-linking method still have trace residues in the hydrogel and cannot be eliminated entirely. When applied in the biological field, it will lead to cytotoxicity, so the development of the entire chemical hydrogel in the medical field is limited.

### 2.3. Physical–Chemical Cross-Linking Hydrogels

In recent years, researchers have tried to combine two kinds of cross-linking methods, the first network adopting physical cross-linking preparation and the second network using chemical cross-linking; the two interpenetrating network structures ensure the mechanical properties of the hydrogel and self-healing.

**Figure 4:**
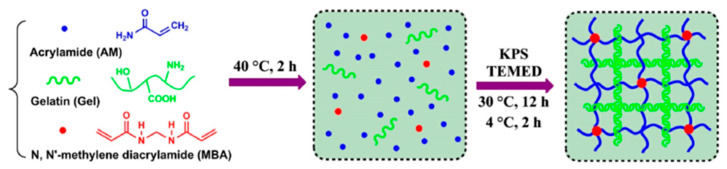
Preparation mechanism of polyacrylamide/gelatin (PAM-gel) hydrogel Reprinted with permission from [19]. 2021, Elsevier.

Chen [25] and others oxidized graphene (GO) and boric acid salt ions into the PVA chain, built the double cross-linking of PVA/borax physical chemistry/graphene oxide hydrogel, hydrogen bond, and boric acid ion stimulation PVA chain and the interaction between components, and the structure of the hydrogel provided a good platform for compressive fatigue resistance. By comparing the amount of GO, it can be concluded that the higher the amount of GO, the more stress the hydrogel can withstand and the better the self-healing and stability. Fan [19] prepared polyacrylamide/gelatin (PAM-gel) hydrogel using the one-pot method (Figure 4), and the mechanical strength of the hydrogel reached 0.57 MPa through the thermal reversibility physical network of gelatin and the chemical cross-linking stability network of polyacrylamide, and PAM-gel hydrogel showed good shape memory function under warm water.

When preparing hydrogels, temperature-sensitive materials are added to give the hydrogels temperature-sensitive characteristics. Generally, hydrogels can be divided into two categories: one is at the highest critical temperature (UCST), and the hydrogels show shrinkage behavior at the lower temperature and the other is at the lowest required temperature (LCST), and the hydrogels show condensation behavior at the higher temperature. This characteristic of hydrogels can be applied in wound dressings. Still, the current difficulty is that the mechanical strength of hydrogels is not enough to achieve the required tensile strength and toughness. Abdolmaleki [26] synthesized super hydrophilic Poly(tris(hydroxymethyl) meth) methacrylamide)/polyvinyl alcohol (PTHMMA/PVA) hydrogel. It can show drastic expansion changes when stimulated by an external environment. The combination of super hydrophilic PTHMMA and PVA’s unique physical and chemical characteristics gives the hydrogel super mechanical properties presented by physical cross-linking and thermal sensitivity of chemical cross-linking.

As reported by Ai [20] et al., in the Xylan/PVA/borax double-network hydrogel prepared using the one-pot method, the hydrogen bond and related tangles between the PVA chain and hydroxyl group on Xylan were used as cross-linking points to construct the first network. The reaction between the PVA chain and borax formed a reversible diol-borate bond, which provided a chemical cross-linking network for the hydrogel. The PVA crystal domain generated by the freeze–thaw method constitutes the second physical network. This dual physical–chemical cross-linking hydrogel has good self-healing and mechanical properties, and the unique reversible network structure makes it have the function of free deformation and recycling. In addition, the adhesiveness of hydrogels is required in some fields, but general protein adhesives tend to have low mechanical strength. Hence, hydrogels with high mechanical strength and high adhesion are the focus of some researchers. Guo [27] used Bovine Serum Albumin (BSA) and polyacrylamide to prepare DN hydrogel. Through physical interaction and chemical covalent cross-linking, such as hydrogen bond and chain entanglement, the hydrogel has good mechanical properties and adhesion.

### 2.4. Radiation Cross-Linking Hydrogels

**Figure 5:**
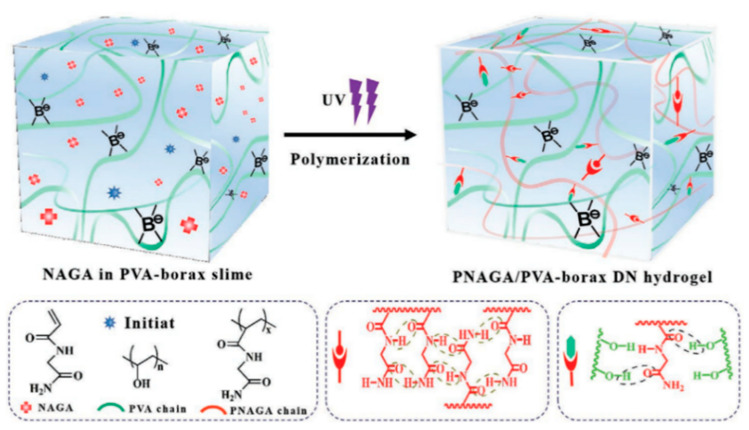
Design and preparation diagram of PNAGA/PVA-Borax (PNAGA/PN-X/PB) DN hydrogel. Reprinted/adapted with permission from [21]. 2020, Wiley.

In 1999, researchers proposed preparing hydrogels using radiation cross-linking. In forming hydrogels, ionizing radiation does not need to add any initiator, cross-linking agent, etc. Because of the participation of radiation technology, hydrogel preparation and sterilization can be carried out at the same time, which avoids the generation of toxic substances [28]. Salmawi [29] used radiation-induced cross-linking to prepare PVA/CS hydrogel, which has an excellent ability to resist microorganisms when applied to wound dressings. Still, the prepared blend is fragile and has poor toughness. To improve the performance of hydrogels after irradiation, Guo [30] immersed PVA/CS hydrogels in tannic acid (TA) solution to prepare PVA-based double cross-linking composite hydrogels, including physical cross-linking between PVA, CS, and TA and chemical cross-linking induced by gamma radiation. Compared with PVA hydrogels prepared by pure radiation, the tensile strength and elongation at the break of the composite hydrogels prepared by the double network mechanism were significantly improved. In recent studies, Ma [21] prepared double cross-linked DN hydrogels based on UV-initiated in situ polymerization of *N*-acryloyl glycinamide (NAGA) in PVA/borax mucilage, as shown in Figure 5. Under UV irradiation, NAGA underwent in situ polymerization, and a double network was constructed between poly(*N*-acryloyl glycinamide) (PNAGA) chains and PVA molecules through hydrogen bonding interactions and dynamic boronic acid bonding. The dynamic double network and effective energy dissipation, especially the gels with a high content of PNAGA, have high mechanical strength, high toughness and fatigue resistance, and the combination of dynamic boronic acid bonding and hydrogen bonding gives the gels good self-healing properties. Chen [31] prepared high-strength PVA/*N*,*N*-Dimethyl-*N*-(3-methacrylamidopropyl)-*N*-(3-sulfopropyl) ammonium betaine (MPDSAH) hydrogel, combining UV irradiation cross-linking and the freezing–thawing method. With the increase in MPDSAH content and irradiation time, the tensile properties of hydrogel with a 15% concentration of MPDSAH increased more than twice compared with a 0% concentration. Moreover, the structure and properties of the hydrogel became more stable due to the blending of MPDSAH and PVA, and the friction coefficient was reduced by 50%. The wear was minor in the friction experiment.

## 3. Mechanical Properties of Double Network Hydrogels

At present, the research on hydrogel preparation has found ten million kinds. According to the research focus in recent years, materials are reviewed, with PVA, alginate(Alg), and protein for basal hydrogel preparation on improving mechanical properties research and the factors that affect performance, including preparation methods, material composition, structure, and the use of the cross-linking agent are analyzed.

### 3.1. Polyvinyl Alcohol Hydrogels

PVA is a water-soluble polyhydroxy polymer. It has non-toxic, environmental protection, and biodegradable properties, and therefore, it is widely used in the areas of medical biology. There are a large number of hydroxyl groups in the PVA molecules, enabling chemical or physical cross-linking hydrogel preparation; PVA hydrogel microcrystal in physical cross-linking, the formation of the three-dimensional network plays an important role in improving the mechanical performance. In the freezing process, the compactness of the internal network of the hydrogel is enhanced, and the relative crystallinity becomes higher. Therefore, it is found that the strength of PVA hydrogel changes with the freezing time and the number of cycles [32].

The mechanical strength of single PVA hydrogel is still not up to the expected effect in some applications [33,34], so taking PVA as the first network and introducing the second network material to prepare double network hydrogel has become a research hotspot. Once tannic acid was introduced into PVA hydrogel and prepared high-strength hydrogel with dimethyl sulfoxide and water as solvents. Hydrogen bonds were formed between the hydroxyl group of the PVA molecule and the phenolic hydroxyl group of tannic acid to control the content of tannic acid. The mechanical strength of the hydrogel showed a trend of first increasing and then decreasing. The tensile strength of the hydrogel reached the maximum value of 2.12 MPa. On this basis, when the PVA/TA hydrogel is immersed in normal saline and the TA concentration is 30%, the maximum tensile strength of the hydrogel can reach 16 MPa, and the mechanical strength is increased by 654%. This is because in the hydrogel structure when hydrogen bonds are generated between molecules, the interaction of normal saline is used as a “sacrifice domain” to dissipate energy. The hydrogel has high tensile strength [35].

### 3.2. Alginate Hydrogel

**Figure 6:**
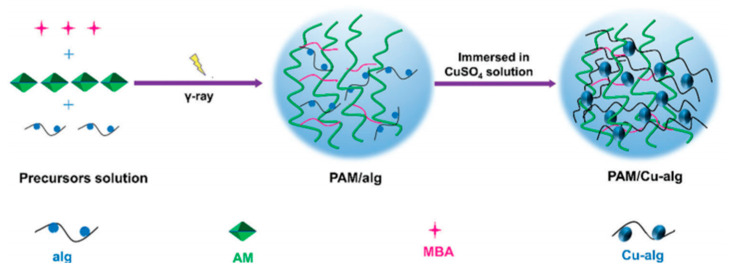
Preparation mechanism of polyacrylamide/copper alginate (PAM/Cu-Alg) double-network hydrogel Reprinted with permission from [38]. 2021, Wiley.

Alginate (Alg) is a kind of natural polymer consisting of *β*-1, 4-D-mannan acid (M) and *α*-1, 4-L-glucuronic acid (G), and owing to its good biocompatibility, is widely used in medical and food fields. Alginate is easy to dissolve into hydrogel with Ca^2+^, and Ca^2+^ is easy to combine with other 2-valent cations in the G region, which also provides a way to solve the problem of poor mechanical properties of hydrogels. In the PAM/Alg system, complete cross-linking of PAM before ion exchange of Alg will lead to poor solvent exchange, and the two polymers will produce micron-scale aggregation. After the water absorption process, hydrogen bonding and metal–ligand coordination stabilize the newly formed alginate aggregates, resulting in a large-scale cross-linking zone. It is helpful to improve the mechanical properties of hydrogels [36]. Zhao [37] found that the hydrogen bond interaction in SA/PAM hydrogel promoted SA self-assembly in the porous matrix of PAM, and the layered semi-interpenetrating network structure also improved the mechanical properties of the hydrogel. Zhang [38] used radiation technology and Cu^2+^ cross-linking to prepare polyacrylamide/copper alginate (PAM/Cu-Alg) double-network hydrogel (Figure 6) to analyze and study the alginate concentration, Cu^2+^ concentration, and irradiation absorbed dose. The ionic interaction between Alg and Cu^2+^ and the hydrogen bond between PAM chains dissipate a large amount of energy, so the degree of Alg cross-linking with Cu^2+^ has a significant influence on the mechanical properties of the hydrogel, and the conductivity of the hydrogel will also be improved because of the addition of Cu^2+^. In addition, research has pointed out that without the addition of SA into polyethylene glycol (PEG) (meth), acrylate hydrogel for specific mechanical properties showed a poor state, from 0% to 2% when the content of the SA was added, and hydrogel compression modulus, strength, failure strain, and toughness had an apparent rising trend. Thus, the SA network to join, combined with the covalent network of PEG (meth) acrylate, the performance of the hydrogel improved to a certain extent [39].

Samp [40] added a small amount of SA to gelatin, which significantly improved the toughness of the hydrogel, but the toughening effect was limited. Young’s modulus of the hydrogel was 3.21 KPa, which was at a low level. Wen [41] adopted the combination of the enzymatic method and ion cross-linking way, with the gelatin network cross-linking by glutamine transferase (TG) as the first network and the alginate network cross-linking by calcium ions as the second network. With the participation of TG and calcium ions, the mechanical properties of the hydrogel were significantly improved. In the latest study, the SA/gelatin hydrogel constructed by Wang [42] changed the original method of metal ion cross-linking and adopted an insoluble alginate crystal domain for cross-linking. Compared with pure gelatin, the water content of the hydrogel was increased by 79%, and Young’s modulus was as high as 0.28 MPa.

### 3.3. Protein-Based Hydrogels

Polymer hydrogels are divided into natural polymer hydrogels and synthetic polymer hydrogels. PVA, SA, and most current hydrogels are all synthetic polymer hydrogels. Although synthetic polymer hydrogel has good mechanical properties and vigorous swelling performance, the nature of the material, its biodegradability, and potential toxicity, to some extent, limit the development of the hydrogel. In recent years, researchers have proposed processes and applications for preparing natural polymer hydrogels, such as protein-based hydrogels.

**Figure 7:**
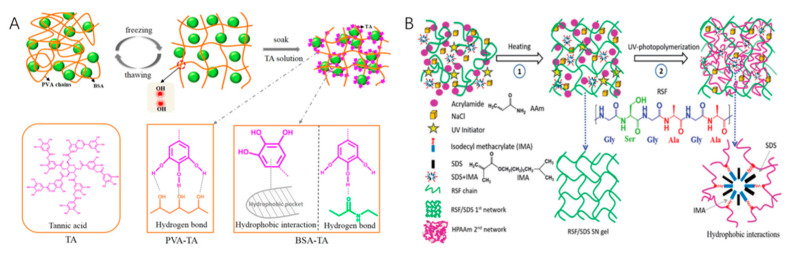
(**A**) Preparation mechanism of TA-PVA/BSA hydrogel Reprinted with permission from [43]. 2018, American Chemical Society; (**B**) Preparation mechanism of RSF/HPAAm hydrogel Reprinted with permission from [45]. 2019, Royal Society of Chemistry.

As a natural macromolecular substance, protein has excellent biocompatibility and degradability. Therefore, in some biomedical application fields, protein-based hydrogels have also attracted the attention of scholars. Pure protein hydrogels have good biocompatibility and uniform structure, but their mechanical properties are poor, and the single protein network also limits the stability and mechanical properties of hydrogels. To solve this problem, combining proteins and polymers has been proposed, using physical and chemical cross-linking to prepare novel protein–polymer hydrogels with a fine structure and desirable properties. Xu [43] integrated BSA into PVA and then freeze–thawed it to form the first network. TA is cross-linking with the BSA protein and PVA chain to create the second cross-linking network based on hydrogen bond and hydrophobic interaction. In this composite hydrogel, the pure physical cross-linking network (TA-PVA/BSA), by controlling the concentration of TA and BSA, the tensile properties can be changed (Figure 7A). By connecting the dimer fiber with a fluorophore to the chemical enzyme of the protein, the hydrogel not only improves the mechanical properties but also has the mechanical chromic function [44].

Silk fibroin (SF) is a natural protein biopolymer. Hydrogels based on SF are mainly prepared using physical/non-covalent cross-linking and chemical/covalent cross-linking. Hydrogen bonds in SF hydrogels stabilize β-sheet structures, which are generally reinforced by inorganic particles or nanofibers [46] and double-physicochemical cross-linking, as well as by blending with other polymers, building double networks and cross-linking at low temperatures to improve their mechanical strength. Chen [45] developed a double-network hydrogel composed of regenerated silk fibroin (RSF) and HPAAm (Figure 7B). RSF/(sodium dodecyl sulfate) SDS hydrogel was used as the first network, and physically cross-linked HPAAm was used as the second network. Both networks were entirely physical structures with great scalability and rapid self-recovery. The compressive strength and tensile strength of the hydrogel reached 122 MPa and 1.17 MPa, respectively. Due to the addition of SDS and sodium chloride in the preparation process, the hydrogel showed good electrical conductivity. The monoclinic structure of silk fibroin gave the microfilaments formed by silk fibroin muscular mechanical strength in water but poor tensile strength and flexibility. Therefore, Ma Mingyu [47] prepared HEMA/SF dual-network hydrogel through in situ polymerization, with silk fibroin as the first network and hydroxyethyl methacrylate (HEMA) as the second network, which has not only high modulus and high-stress response under high strain conditions, but also has certain high deformation and fatigue resistance.

### 3.4. Other Types of Hydrogels

Natural starch is a typical bio-based polymer. It consists of 20–25% amylose and 75–80% amylopectin, the internal structure of the species contains a large number of hydroxyl groups, so it has a solid cross-linking ability. Studies have shown that the toughness of DN hydrogel prepared using polyvinyl alcohol/starch can reach 290.5 KJm^−3^ and the compressive strength can reach 547.8 KPa [48]. Carboxymethyl starch (CMS), as a starch derivative, is a kind of water-soluble anionic polymer that is widely used in the food and paper industry. Liu [49] and others prepared CMS DN hydrogels, with the first network using covalently cross-linked PAM and the second network using CMS of carboxyl and Fe^3+^ dynamic physical coordination. The fracture stress of the hydrogel was 483 ± 28 KPa, and the Fe^3+^ can be used as a high-sensitivity stress sensor for mechanical response due to the migration of Fe^3+^ in the porous structure inside the hydrogel.

As an alkaline polysaccharide, chitosan has excellent antimicrobial properties; its long chain structure is not easily dissolved in water and has a high viscosity, and low molecular weight chitosan easily dissolved in water and is easy to produce all kinds of rigid and brittle networks of chitosan. As a result, Yang [1] and others built the first network with high viscosity and high energy dissipation with low molecular weight chitosan; polyacrylamide acts as a secondary network to construct a soft and pliable network. The energy dissipation mechanism of chitosan works in synergy with the covalent linkage of polyacrylamide to enable complex bonding of the hydrogel with unorganized materials and different metals, with interface toughness up to 1375.2 JM^−2^. General water hydrogel, due to its high internal water content, cannot be used in a low-temperature environment. When the hydrogel is in sub-zero temperatures, it begins to produce ice crystals internally, so the hydrogel’s softness, stretchability, and conductivity are affected, and after tests, CS/PAM gel at sub-zero temperatures can still maintain good antifreeze performance.

## 4. Application

### 4.1. Biomedical Applications

**Figure 8:**
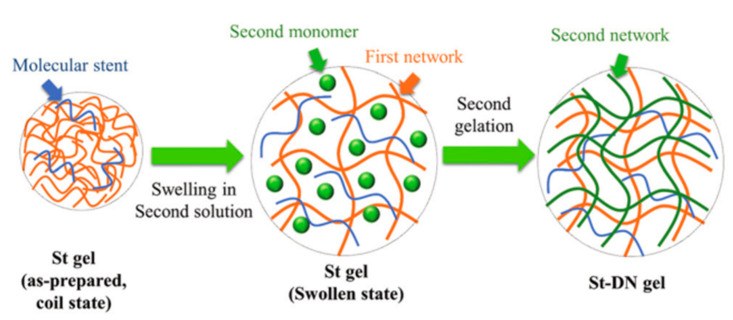
Preparation mechanism of St DN hydrogel. Reprinted with permission from [51]. 2021, Royal Society of Chemistry.

The first network of dual network hydrogels mainly uses strong polyelectrolytes, which also significantly limits the development of DN hydrogels in biomedicine. Therefore, the concept of biomimetic DN hydrogels was proposed to replace strong polyelectrolytes with neutral polymers of “molecular scaffolds” [50], such as poly (*N*, *N*-dimethylacrylamide) (PDMAAm). This neutral polymer constructs the first and second networks and has good biocompatibility. Meanwhile, some biopolymers, such as chondroitin sulfate proteoglycan or sodium hyaluronate, act as molecular scaffolds. The constructed scaffold DN (ST-DN) hydrogel (Figure 8), compared with the traditional DN hydrogel, has not only good mechanical properties but also is conducive to cartilage regeneration [51]. The carboxymethyl hydrogel polysaccharide (CMCD)/PAAm hydrogel was prepared by Wang [52]. The synergistic effect of the double network gives the hydrogel good toughness and comprehensive mechanical properties when the water content is 75%. Through the analysis of the concentration and osmotic stress of the internal components of the hydrogel, it was found that it can meet the required toughness for repairing cartilage. Furthermore, the hydrogel protects the defect and provides the ability to deliver drugs, helping cartilage regenerate quickly. Other soft tissues, such as ligaments, tendons, and menisci, can disperse stress after undergoing large deformations. As a hydrogel of biomimetic articular cartilage, its structure and properties are similar to those of natural articular cartilage. Still, the three-dimensional network disordered structure of hydrogel makes its mechanical properties and tribological properties less than ideal. To solve this problem, Chen [53] prepared a high-strength polyvinyl alcohol-nano-hydroxyapatite-polyacrylic acid (PVA-HA-PAA) composite hydrogel through directional stretching, freeze–thawing, and annealing. The addition of different concentrations of HA significantly improved the mechanical properties and toughness of PVA/PAA hydrogel. When the sliding direction was parallel to the tensile direction, the friction properties of anisotropic hydrogels were significantly better than those of vertical and isotropic hydrogels. Human tendons are reinforced bundles to ensure their function, and hydrogels are of great significance in tendon simulation due to their excellent biocompatibility. Luo [54] prepared PVA/TA hydrogel based on the simple strategy of PVA and TA, and the hydrogen bond between PVA and TA dissipated energy. The toughness and mechanical properties of the hydrogel are better than those of most hydrogels. Moreover, the hydrogel immersed in deionized water, phosphate buffer solution (PBS), and simulated body fluid (SBF) solution for seven days had the same strength and volume as before immersion, which effectively helped the tendon to recover.

In human muscle, some ions, such as sodium, magnesium, and potassium, can change the mechanical properties of power under certain conditions. Therefore, inspired by this, researchers have tried to assign ionic properties to the structure of hydrogels in the past few years to change the ions in the hydrogel so that it has a certain ionic response performance. The water content of hydrogels can be controlled according to the osmotic pressure of ions. When hydrogels are immersed in solutions with the same salt concentration but different ions, the difference in osmotic pressure leads to differences in the hardness, flexibility, and toughness of hydrogels [55]. Inspired by the muscle structure, Geng [56] prepared PAM-co-acrylic acid (AA)/cellulose nanofiber (CNF) /Fe^3+^ dual-network hydrogel with a layered system. The internal system provides sufficient energy-dissipation and the ion channels provided by aligned CNF, so the hydrogel has not only strong mechanical properties but also good ionic conductivity.

Self-healing hydrogels can realize self-repair after damage healing function. This is due to the physical reversible hydrogen bonding of hydrogel, which is widely used in the biological tissue engineering field. The study found that most of the hydrogel, because of high water content and the brittleness of the network, the self-healing process, before and after its mechanical performance, will also change. Therefore, self-healing hydrogels are still a significant challenge in terms of durability and stability. The self-healing hydrogel preparation strategy proposed by Tavakolizadeh [57] group found that the hydrogen bond between oxidized SalEP (Osa) and ethylenediamine modified SalEP (SaHEA) chain (OSEA network) is cross-linking with Schiff base to realize the self-healing ability of the hydrogel. The mechanical strength and toughness of the hydrogel are learned through the interaction of hydroxyl groups in the PVA network, and Young’s hydrogel modulus is up to 14 KPa. The ability to heal itself can recover more than 98% within 21 days, which provides an excellent option for helping skin tissue regenerate. In addition, supramolecular polymers formed by the host–guest interaction can be automatically connected without external stimulation after network disconnection. This has also been applied to improve the self-healing property of hydrogels. The team of Li [58] introduced chitosan into reactive carboxyl groups, sulfur groups, and cyclodextrins, which are common groups for constructing hydrogels based on host–guest interactions, and secondly carboxyl groups also improved the water solubility of chitosan and also created favorable conditions for constructing DN hydrogels. High cross-linkage also led to improved performance of hydrogels, and dynamic non-covalent bonded host–guest interactions also played a dominant role in the self-healing properties of hydrogels.

### 4.2. Application of Intelligent Sensors

With the improvement in living standards, people have begun to pay more and more attention to wearable electronic devices, which are used to detect their health status and movement status. The mechanism is that the mechanical deformation of sensors changes parameters such as resistance and capacitance and changes the automated signals into electrical signals. Intelligent hydrogels can respond to external stimuli, while traditional elastic strain sensors are not sensitive enough in the case of large deformation. Therefore, the solid tensile strength and flexibility of DN hydrogel can solve the shortcomings, and it has a unique attraction in flexible sensors [59]. Both traditional hydrogels and ionic liquid-based hydrogels have high internal water content [60]. When the temperature rises, the evaporating water and leaking ions affect the electronic devices. Therefore, a liquid-free dual network ionic conductor (LFDNIC) was proposed by Zhao [61]. It comprises a stretchable poly (AA-ChCl) type supramolecular deep eutectic polymer and a brittle polyvinylpyrrolidone (PVP). Dynamic hydrogen bonding ensures the mechanical properties of the hydrogel, and the electrical conductivity of the hydrogel is also enhanced by the addition of ionic conductors. By modifying SA, the formation of methacrylic acid glycidyl esterified sodium alginate (GMA-SA), and then added acrylamide, the preparation of GMA-SA-PAM hydrogel, compared with the unmodified sodium alginate prepared SA-PAM hydrogel. Its mechanical properties increased by 171.18%, which is due to the formation of an interoperability network and the increase in cross-linking density so that the mechanical properties and toughness of the hydrogel have improved. In terms of conductivity, the GMA-SA-PAM hydrogel is immersed in NaCl to form a GSP-Na hydrogel, and the internal mesh structure and high content of metal ions give the hydrogel its conductivity and through electrochemical testing, the conductivity of GSP-Na hydrogel reaches 5.26 Sm^−1^, which also exceeds most semiconductor materials [62].

### 4.3. Application of Ion Adsorption

It is self-evident that water plays a vital role in nature and human society. The development of the industry is also inseparable from water resources. In some industries, such as the paper industry, dyeing and weaving industry, and plastic industry, the heavy metals (Pb, Ni, Cd, Cu) in water exceed the standard, which is a significant problem becoming more and more serious. In recent years, the use of hydrogels for ion adsorption has become the mainstream research direction, such as cation adsorption [63] and molecular imprinting [64]. Researchers have found that the adsorption function of gels with dual network structures has been further enhanced. For example, the adsorption capacity of polyacrylic acid/humic acid (PAA/HS) DN gel prepared using Ma [65] based on humic substances for Cd (ii), Cu (ii), and Pb (ii) was 412.76, 151.00, and 360.5 mg/g, respectively. Song [66] prepared carboxymethyl cellulose/polyethyleneimine (CMC/PEI) DN gel using a one-step method, and the adsorption capacity of Cr (VI) was 6.01 mmol/g.

However, as adsorbents, hydrogels may be in groundwater, which will be subjected to pressure from soil and water flow. Long-term loading is a challenge to the mechanical properties of hydrogels. Cao [67] changed the concentration of SA in PAM/SA/Fe^3+^ DN gel and found that different concentrations of SA had various adsorption capacities for ions. When the concentration of SA was 10.3%, the maximum adsorption capacity of the gel for methylene blue could reach 44.02 mg/g. Compared with PAM/SA gel (full compressive strength 0.152 MPa), the full compressive strength of PAM/SA/Fe^3+^ can reach 0.778 MPa. Fei [68] studied the removal of heavy metal ions in water based on silica/polyacrylic acid dual-network gel. When the concentration of copper ions was 1200 mg/L, the adsorption capacity of copper ions on the gel could reach 700 mg/g within 2 h, and the formation of a silica network in the gel was helpful in improving the mechanical strength of the gel. Tang [69] prepared gels by physical cross-linking of chitosan (CTS), SA, and calcium ions (Ca^2+^), which consisted of the first network CTS/SA, and the second network SA/Ca^2+^. In terms of performance, the maximum tensile strength of the hydrogel is 0.19 MPa, and the gel has good adsorption performance due to the electrostatic interaction inside the gel and the coordination of nitrogen atoms.

## 5. Conclusions

This paper reviews the research on the mechanical properties of DN hydrogels in recent years. Compared with single-network hydrogels, the energy dissipation of dual-network structures and the role of “sacrificial bonds”, the mechanical properties of DN hydrogels are also improved. DN hydrogels have been developed for nearly 20 years, and now they have been applied in various fields. Representative polyvinyl alcohol DN hydrogels and alginate DN hydrogels have been used in biomedicine, intelligent sensors, ion adsorption, and other fields. Their biocompatibility and self-healing properties have also been widely studied. Given the current development of DN hydrogels, there are still several difficulties in future research. (1) DN hydrogels should be combined with other polymers, such as photonic crystals, while satisfying high mechanical properties, which will be helpful for the study of ion adsorption capacity and mechanical force chromaticity. (2) The ion adsorption capacity of DN hydrogel is mainly applied to metal ions at present, and it should be studied in the field of adsorbing gas pollutants in the future. (3) The stimulation of multiple responses should be more accurate and sensitive to adapt to the rapid development of intelligent sensors.

## Figures and Tables

**Table 1:** Differences in mechanical properties of hydrogels prepared by different cross-linking methods.

Cross-Linking Method	Hydrogel Name	First Network	Second Network	Tensile Strength	Ref.
Physical cross-linking	Agar/Hydrophobic associated polyacrylamide hydrogels	Agar hydrogel network	Hydrophobically associated polyacrylamide gel network	0.267 MPa	[15]
	Gelatin/Polyacrylic acid/Tannic acid/Aluminum chloride hydrogels	Gelatin, polyacrylic acid, tannic acid hydrogen bonding network	Al^3+^ ligand bonding network with polyacrylic acid	216 KPa	[16]
	Chitosan/Polyacrylic acid/Ag^+^ hydrogels	Chitosan/Ag^+^ cross-linking network	Polyacrylic acid network	24 MPa	[17]
Chemical cross-linking	Poly(2-acrylamido-2-methylpropanesulfonic acid)/Poly (*N*-isopropyl acrylamide-co-polyacrylamide) hydrogels	Poly(2-acrylamido-2-methylpropane sulfonic acid) network	poly(*N*-isopropyl-acrylamide-co-acrylamide) network	25 MPa	[18]
Physical-chemical cross-linking	Polyacrylamide/Gelatin hydrogels	Gelatin network	Polyacrylamide network	0.57 MPa	[19]
	Xylan/PVA/Borax hydrogels	PVA chain with Xylan first physical network, PVA crystal domain second physical network	PVA chain and borax chemical network	81 KPa	[20]
Radiation Crosslinking	*N*-Acryloyl glycinamide/PVA-borax hydrogels	PVA/Dynamic boronic acid lipid bond	poly(*N*-acryloyl glycinamide)/PVA network	200 KPa	[21]

## Data Availability

Not applicable.
